# Regulation of otic neurosensory specification by Notch and Wnt signalling: insights from RNA-seq screenings in the embryonic chicken inner ear

**DOI:** 10.3389/fcell.2023.1245330

**Published:** 2023-10-12

**Authors:** Magdalena Żak, Thea P. Støle, Vincent Plagnol, Nicolas Daudet

**Affiliations:** ^1^ UCL Ear Institute, University College London, London, United Kingdom; ^2^ Genetics Institute, University College London, London, United Kingdom

**Keywords:** Notch signailing pathway, Wnt/β-catenin signaling pathway, RNA-seq-RNA sequencing, prosensory specification, inner ear development, chicken embryo

## Abstract

The Notch and Wnt signalling pathways play key roles in the formation of inner ear sensory organs, but little is known about their transcriptional effectors and targets in this context. Here, we perturbed Notch and Wnt activities in the embryonic chicken otic vesicle using pharmacological treatment or *in ovo* electroporation of plasmid DNA, and used RNA-Seq to analyse the resulting changes in gene expression. Compared to pharmacological treatments, *in ovo* electroporation changed the expression of fewer genes, a likely consequence of the variability and mosaicism of transfection. The pharmacological inhibition of Notch activity induced a rapid change in the expression of known effectors of this pathway and genes associated with neurogenesis, consistent with a switch towards an otic neurosensory fate. The Wnt datasets contained many genes associated with a neurosensory biological function, confirming the importance of this pathway for neurosensory specification in the otocyst. Finally, the results of a preliminary gain-of-function screening of selected transcription factors and Wnt signalling components suggest that the endogenous programs of otic neurosensory specification are very robust, and in general unaffected by the overexpression of a single factor. Altogether this work provides new insights into the effectors and candidate targets of the Notch and Wnt pathways in the early developing inner ear and could serve as a useful reference for future functional genomics experiments in the embryonic avian inner ear.

## 1 Introduction

The inner ear, implicated in sound and equilibrium perception, has a highly elaborate three-dimensional architecture. Its dorsal part, the vestibular system, contains five sensory organs sensitive to head position and movements: the utricle, the saccule, and three semi-circular canals and their associated cristae. In its ventral aspect is the cochlear duct, which contains an auditory epithelium called the organ of Corti in mammals, or the basilar papilla in birds and reptiles. All sensory epithelia of the inner ear contain specialised mechanosensory “hair” cells and their supporting cells, arranged in a salt-and-pepper pattern. In response to the deflection of their stereocilia, induced by sound or head movements, the hair cells release neurotransmitters at their synaptic pole and stimulate the auditory and vestibular neurons that innervate them.

The molecular mechanisms of hair cell formation and their associated neurons are under intense scrutiny, given their relevance to the diagnosis and treatment of the most common forms of congenital and progressive hearing loss in humans. The vast majority of the cells that compose the inner ear derive from the otic placode, an ectodermal derivative located on both sides of the embryonic hindbrain (Basch et al., 2016). The placode invaginates and closes itself to form the otic vesicle, or otocyst, which then undergoes a rapid growth and 3-dimensional transformation to form the various sensory and non-sensory epithelial compartments of the inner ear. The hair cells, supporting cells, and neurons of the cochleo-vestibular ganglion derive from neurosensory-competent cells that are specified within the ventro-medial wall of the otic vesicle (Adam et al., 1998; Morsli et al., 1998; Fritzsch et al., 2002; Satoh and Fekete, 2005; Mann et al., 2017; Steevens et al., 2017). The common progenitors for hair cells and supporting cells, called “prosensory” cells, express the transcription factor SOX2, which is required for the formation of all sensory organs (Kiernan et al., 2005; Neves et al., 2007; Pan et al., 2013). SOX2 is initially expressed along a broad ventral domain extending along the antero-posterior axis of the otic vesicle, before its restriction to two prosensory domains at its anterior and posterior poles (Steevens et al., 2017). The posterior patch gives rise to the posterior crista only, whilst the anterior domain expands and splits into distinct vestibular organs (Mann et al., 2017). The anterior prosensory domain is also neurogenic: otic neuroblasts upregulate the proneural factors Neurogenin1 and NEUROD1 and delaminate from this SOX2-expressing domain before differentiating into vestibular and auditory neurons (Steevens et al., 2017).

Besides these cell-intrinsic factors, two important cell-to-cell communication pathways regulate the spatial and temporal progression of neurosensory specification in the early developing inner ear: Notch and Wnt signalling (Żak et al., 2015; Daudet and Żak, 2020). Notch signalling depends on direct cell contact between cells expressing transmembrane ligands of the Delta/Jagged family and Notch receptors (Daudet and Żak, 2020). In canonical Notch signalling, the binding of a ligand to the extracellular domain of a NOTCH receptor triggers a series of proteolytic cleavages, catalysed by gamma-secretase and Adam proteases, that release the intracellular domain of NOTCH (NICD), which then translocates to the nucleus to regulate gene expression (Daudet and Żak, 2020). The Notch pathway plays two critical roles in the early specification of the neurosensory cells of the inner ear (Daudet and Żak, 2020). First, it acts by lateral inhibition to regulate otic neurogenesis: the neuroblasts express the ligand Delta-like 1 (DLL1), which drives Notch activity in neighbouring cells to repress proneural gene (and DLL1) expression. Its second role is the maintenance of prosensory specification: the prosensory cells express the Notch ligand Jagged 1 (JAG1), which in this context is positively regulated by Notch activity. This process, called lateral induction, elevates Notch activity and is required for the maintenance of SOX2 expression within prosensory domains.

Wnt signalling, on the other hand, relies on diffusible Wnt ligands that can act at a distance and bind to transmembrane receptors of the Frizzled family (Komiya and Habas, 2008). Wnt signalling can elicit very different intracellular responses, affecting Ca2+ signalling, the planar cell polarity machinery, or gene expression by the “canonical” Wnt/β−catenin pathway. In the latter mode, Wnt activity leads to an elevation of β−catenin intracellular levels, which then interacts with transcription factors of the TCF/LEF family to regulate the expression of specific target genes (Komiya and Habas, 2008). The Wnt/β−catenin pathway has been shown to be essential for the dorso-ventral patterning of the otic vesicle and in particular the morphogenesis of the vestibular system (Riccomagno et al., 2005; Noda et al., 2012). Furthermore, our recent work has shown that Wnt activity controls SOX2 expression in a dose-dependent manner in the otic vesicle: high levels of Wnt repress SOX2 in the dorsal part of the otic vesicle, thereby restricting neurosensory competent domains to its ventral aspect (Żak and Daudet, 2021).

Given the prominent roles of Notch and Wnt signalling in the specification of the neural and prosensory cells of the inner ear, we sought to identify their transcriptional targets in this context. We performed various pharmacological and genetic manipulations of both pathways in the embryonic chicken inner ear and analysed the resulting changes in gene expression using RNA-seq. The results of our bioinformatics analyses suggest that a large set of genes associated to neurosensory specification are regulated by the Notch and Wnt pathway and revealed potential nodes of interactions between these pathways. There were however some limitations in terms of reproducibility of the results at the individual gene level, possibly arising from the use of bulk cell populations and the chicken embryo as an animal model. We discuss the implications of our findings in relation to the molecular mechanisms of neurosensory specification and some of the lessons learned in terms of experimental design, bioinformatics analysis and functional validation of RNA-Seq screenings in the inner ear.

## 2 Results

### 2.1 Treatment with gamma-secretase inhibitor for 6 h leads to strong perturbation of Notch activity

**FIGURE 1:**
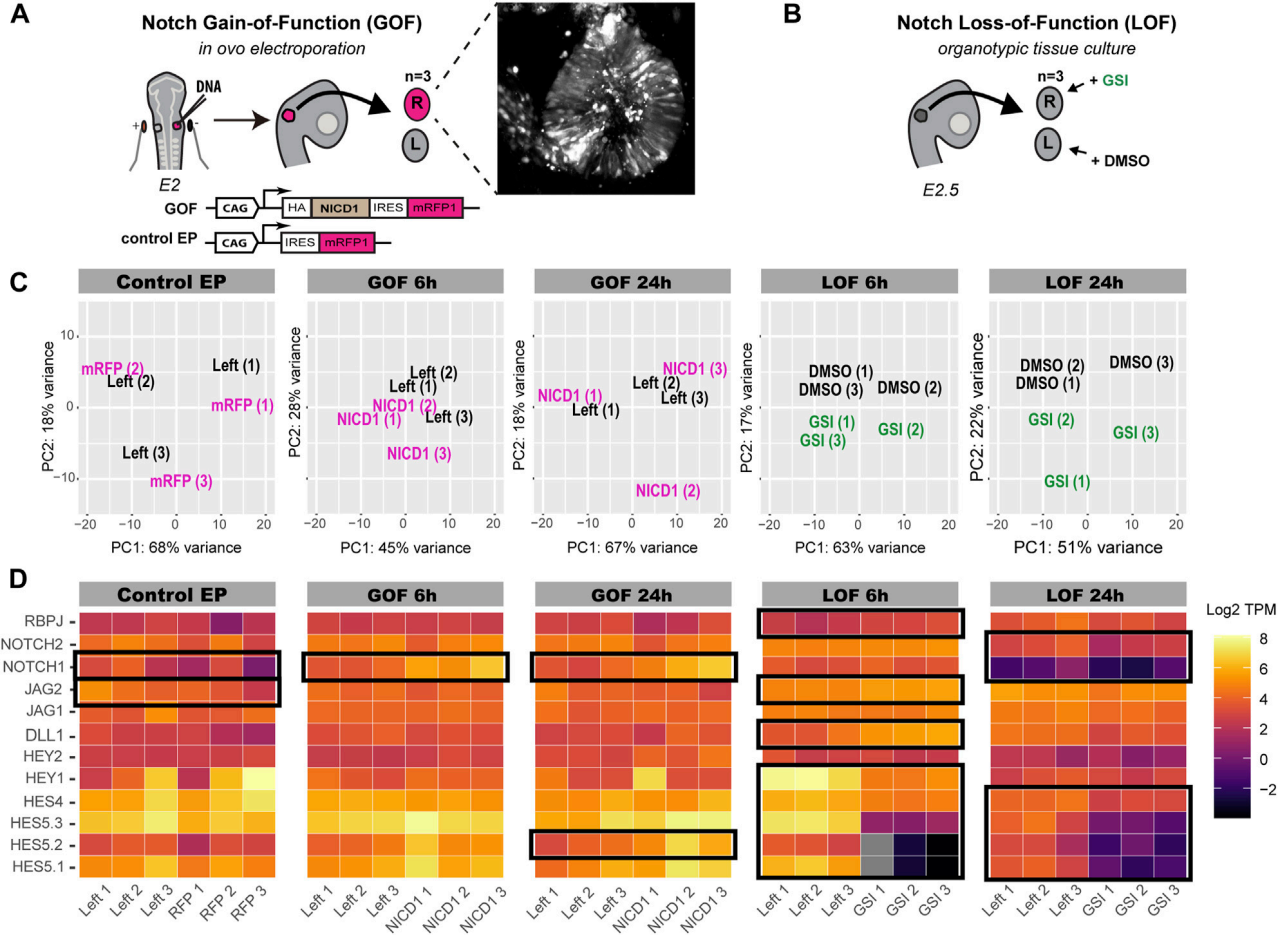
Characterisation of Notch GOF and LOF bulk RNA-seq datasets. **(A,B)** Schematics of the experimental design. **(A)** E2 chicken right otic placodes were transfected with either pCAB construct containing NICD1-IRES-mRFP1 cassette (Notch GOF) or pCAB plasmid expressing only mRFP1 (Control EP). Ears were collected after 6 h (Notch GOF 6 h) or 24 h (Notch GOF 24 h, Control EP) and only 3 strongly transfected ears, as shown in an exemplary picture, together with their left counterparts from the same embryo were sequenced. In the Notch LOF **(B)**, 3 pairs of E2.5 chicken otic cups were dissected and incubated in media containing γ-secretase inhibitor (GSI) LY411575 or DMSO for 6 h (Notch LOF 6 h) or 24 h (Notch LOF 24 h). **(C)** PCA plots for each analysed Notch dataset. **(D)** Heatmaps (log TPM) for Notch signalling elements detected by bulk RNA-seq in chicken otocyst. High number of transcripts is marked by light yellow and very low by dark purple. Note that genes which were not expressed and had zero copies of transcripts are shown in grey. Black frames indicate genes, which expression was significantly changed in response to manipulation of Notch activity. Note that *NOTCH1* and *JAG2* were significantly dysregulated in Control EP.

To gain new insights into the transcriptional targets of Notch signalling, we manipulated Notch activity in embryonic chicken otocysts and analysed the resulting changes in gene expression levels with RNA-Seq. To stimulate Notch activity (gain-of-function, or GOF), we electroporated *in ovo* the right otic cup of E2 (stage HH12-14) chicken embryos with a plasmid encoding the intracellular domain of the chicken NOTCH1 receptor (NICD1) (Figure 1A). Control embryos were electroporated with a monomeric red fluorescent protein (mRFP1) expression construct. Next, RNA was isolated from both transfected (right) and untransfected (left) otocysts of 3 embryos for each of the following conditions: Notch GOF 6 h post-electroporation (post-EP), Notch GOF 24 h post-EP, and Control 24 h post-EP. For blocking Notch activity (loss-of-function, or LOF), we treated E2.5 otocysts *in vitro* with the γ-secretase inhibitor LY411575 (GSI) for 6 and 24 h (Figure 1B). Three embryos were used at each time point, with the right otocysts treated with 10 µM LY411575 whilst the left ones were kept in medium supplemented with DMSO at matching concentration as a control. At the end of the treatment, RNA was extracted from each cultured otocyst and processed for RNA-Seq, then analysed using the Kallisto and Sleuth packages for differential gene expression analysis.

To assess the effectiveness of the different treatments, we first generated plots for unsupervised principal component analysis (PCA) for each condition. The left and right (transfected) otocysts from the same embryo clustered together in the control electroporation (control EP) (Figure 1C), suggesting very little or no effects of the electroporation itself. The plots for Notch GOF 6 h and GOF 24 h showed respectively little and no obvious separation between samples electroporated with NICD1 and their left untransfected counterparts, suggesting that the overexpression of NICD1 did not trigger major and reproducible changes in the transcriptomes of transfected otocysts. In contrast, right ears treated with LY411575 and left control ears from the same embryo separated along the PCA2 in the plots for Notch LOF 6 and 24 h, suggesting an effect of the treatment.

To see whether any of the GOF or LOF treatments significantly affected the expression of components of the Notch pathway, we generated heatmaps representing the normalised transcripts abundance of TPM (Transcripts Per Million) for the direct Notch targets and effectors of the HES family (*HES5.1*, *HES5.2*, *HES5.3*, *HES4*, *HEY1*, *HEY2*), Notch receptors (*NOTCH1*, *NOTCH2*) and ligands (*DLL1*, *JAG1*, *JAG2*), and the transcriptional regulator *RBPJ* (Figure 1D, TPM and log TPM values in Supplementary Table S1). Surprisingly, the expression of *NOTCH1* and *JAG2* was significantly decreased after electroporation with the control construct (Figure 1D). In the Notch GOF 6 and 24 h datasets, we detected a significant increase in NOTCH1 expression (q-value < 0.05, Figure 1D). However, further analysis (Supplementary Table S1) in which we modified the reference chicken genome showed that the number of NOTCH1 intracellular domain transcripts was significantly increased (q-value < 0.001), most likely reflecting overexpression of the construct, while the expression levels of the extracellular and transmembrane domain of the endogenous NOTCH1 gene did not change. Of note, none of the *HES* genes were affected in Notch GOF 6 h group and only *HES5.2* increased its expression in the Notch GOF 24 h condition. In contrast, blocking Notch activity with LY411575 for 6 h lead to a significant decrease in the expression of 5 *HES* genes (*HES5.1*, *HES5.2*, *HES5.3*, *HES4*, *HEY1*) and an increase in *DLL1*, *JAG2* and *RBPJ* (raw TPM and log TPM values in Supplementary Table S1). A clear, but not as strong as with the 6 h treatment, reduction in the expression of *HES5.1*, *HES5.2*, *HES5.3*, *HES4*, *NOTCH1*, and *NOTCH2* was also observed after 24 h treatment with LY411575 (Figure 1D). These results show that LY411575 efficiently inhibits Notch activity, and the short treatment (6 h) with LY411575 appears to induce stronger changes in the expression of direct Notch target genes than the 24-h treatment.

**FIGURE 2:**
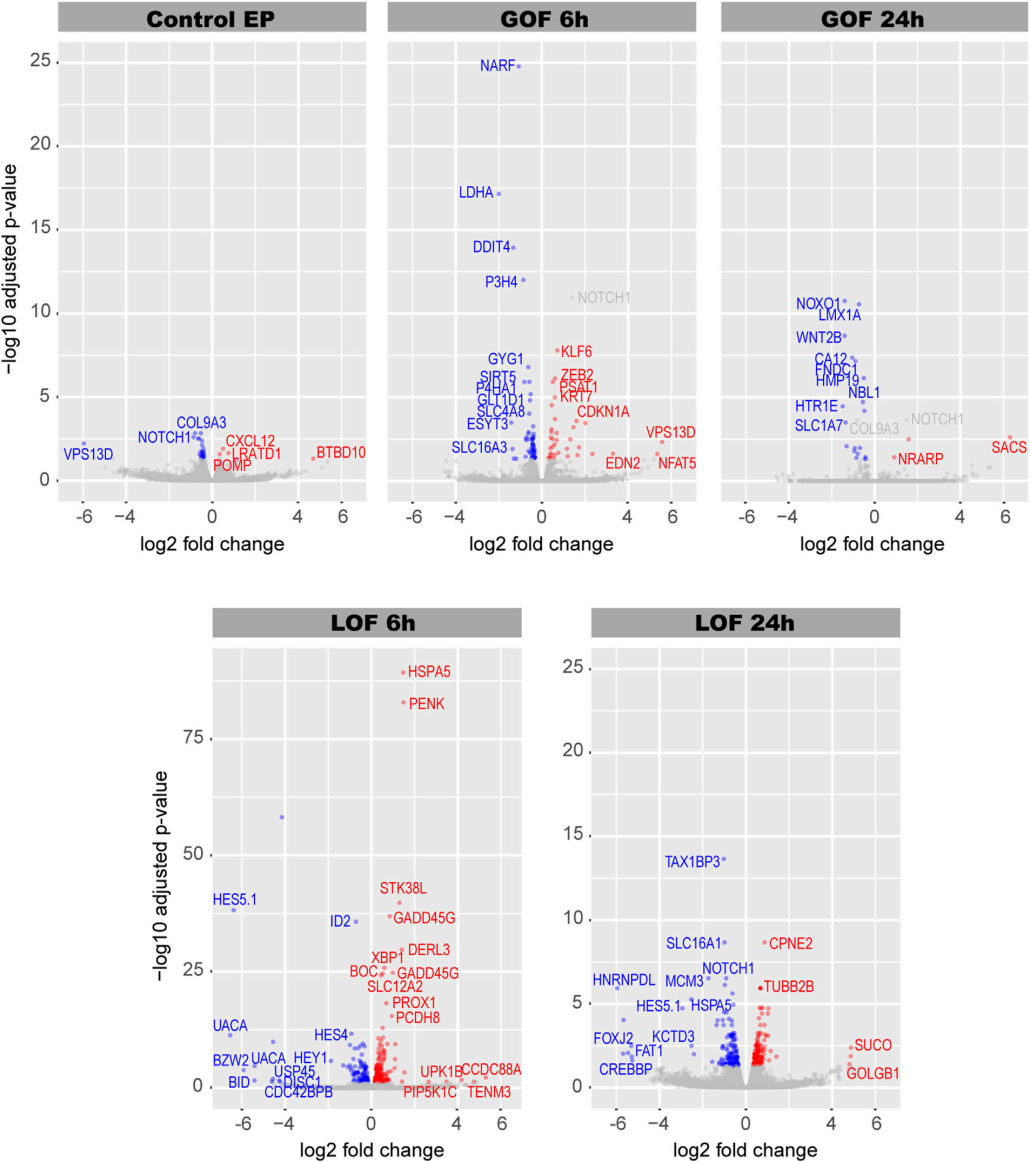
Volcano plots for each Notch dataset. In blue are genes with decreased expression and in red genes with increased expression. Gray dots represent non-significant genes. Additionally, *NOTCH1* and *COL9A3* are also highlighted in gray in Notch GOF 6 h and Notch GOF 24 h Volcano plots because their expression was also significant in Control EP and therefore were excluded from further analysis.

The differential gene expression analysis revealed that 28 genes were significantly (q-value < 0.05) regulated in the control EP samples (Figure 2; Supplementary Table S1); these were removed from the Notch GOF 6 and 24 h datasets in the subsequent analyses. A total of 53 genes were downregulated and 34 upregulated in Notch GOF 6 h condition (Figure 2; Supplementary Table S2), while 19 genes reduced their expression and 3 genes increased in Notch GOF 24 h dataset (Figure 2; Supplementary Table S3). Among genes significantly dysregulated in the Notch LOF 6 h, 105 genes showed a decrease and 181 genes an increase in expression levels (Figure 2; Supplementary Table S4). A comparable result was obtained in the Notch LOF 24 h dataset, with 127 genes downregulated and 128 genes upregulated (Figure 2; Supplementary Table S5). Surprisingly, *SOX2* and *JAG1*, two genes implicated in prosensory specification and presumed to be positively regulated by Notch activity, were not among the significantly regulated genes in any of the datasets.

Altogether, the PCA results and the analysis of the expression of Notch components indicate that the pharmacological treatment with LY411575 leads to a stronger and more consistent perturbation of Notch activity than the *in ovo* electroporation of NICD1.

### 2.2 Genes implicated in neurogenesis and inner ear formation are among the early Notch targets

We next used the ToppGene bioinformatics platform to perform a functional annotation of the differentially expressed genes (q-value < 0.05) of each dataset. The Notch GOF 6 h dataset, with 90 differentially expressed (DE) genes (Supplementary Table S2), contained primarily genes associated with metabolic and general cell biological processes, indicating that this time point was too early to detect Notch-induced changes in expression of classic Notch target genes after electroporation. The GOF 24 h dataset (Supplementary Table S3) contained only 22 DE genes, which is not sufficient for a robust statistical analysis of biological functions or pathway enrichment. Nevertheless, several of the DE genes have been implicated in inner ear development (*LMX1A*, *LMX1B*, *WNT2B*, *BMPER*, *SLITRK6*) (Nichols et al., 2008; Katayama et al., 2009; Sienknecht and Fekete, 2009; Mann et al., 2017).

We present in detail the results obtained for the Notch LOF 6 h and Notch LOF 24 h datasets, which contained a larger number of DE genes, enabling a more robust bioinformatics analysis of their functions.

**FIGURE 3:**
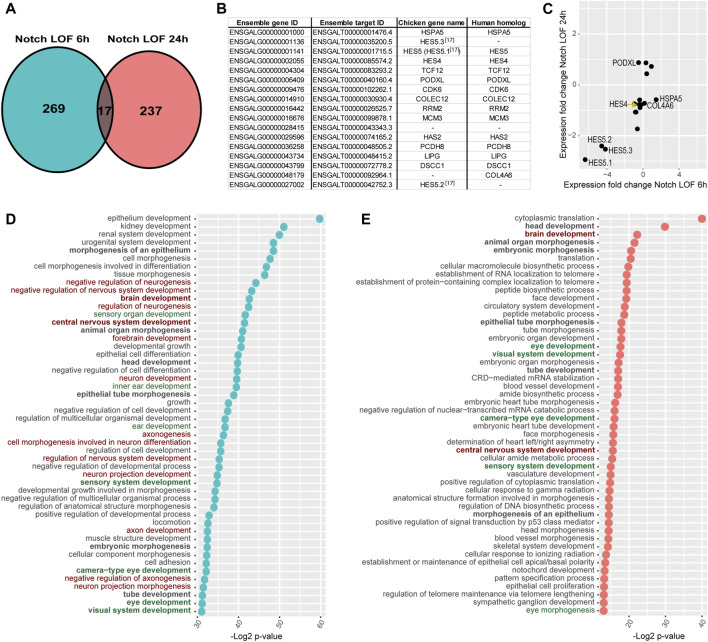
Comparison of Notch LOF 6 h and Notch LOF 24 h datasets. **(A–C)** Characterisation of genes shared between the two datasets. Venn diagrams showing that only 17 genes are in common **(A)** and a table listing the Ensembl gene and target IDs of these 17 genes as well as chicken gene name and its human homolog name **(B)**. The direction of changes in the expression of 14 out of these 17 genes was similar between Notch LOF 6 h and Notch LOF 24 h **(C)**. Only for 3 genes (*PODXL*, *HSPA5*, *COL4A6*) these changes in expression were not aligned. **(D,E)** Top 50 GO Biological Functions identified by TopGene for each of the dataset. In bold are highlighted twelve biological functions shared between Notch LOF 6 h and Notch LOF 24 h. Note that both datasets have several functions associated with inner earand sensory organ development (in green), but Notch LOF 24 h, in contrast to Notch LOF 6 h, has only two linked with neurogenesis and formation of neurons (in red).

The Notch LOF 6h and 24 h datasets shared 17 genes only (Figure 3A), suggesting that the transcriptional response to Notch inhibition varies greatly over time. Reassuringly, these include *HES5.1*, *HES5.2*, *HES5.3*, and *HES4*, which together with *TCF12* belong to the basic helix-loop-helix (bHLH) family of transcription factors regulating neurogenesis (Figure 3B). All the *HES* genes showed a reduced expression in both datasets (Figure 3C) suggesting a sustained inhibition of Notch activity. The remaining genes were: *HSPA5*, *PODXL*, *CDK6*, *COLEC12*, *RRM2*, *MCM3*, *HAS2*, *PCDH8*, *LIPG*, *DSCC1*, *COL4A6*, and two unknown genes (Figure 3B).

Next, we used the ToppGene functional annotation platform to analyse the two datasets. We found that 12 of the top 50 Gene Ontology (GO) functions of each dataset were shared; these include functions related to tissue morphogenesis, embryonic development, and sensory and central nervous system development (Figure 3D; Supplementary Tables S4, S5). In the Notch LOF 6 h dataset, 7 GO functions were associated with ear and sensory organ development and 14 related to the formation of the nervous system and neuronal differentiation (Figure 3D; Supplementary Table S4). In contrast, the Notch LOF 24 h dataset contained mainly genes associated with various cell biological processes and embryonic organ development (Figure 3E; Supplementary Table S5). Among the top 50 GO functions for this dataset, 5 were linked to eye development and only 2 with central nervous system development. Furthermore, the Notch LOF 6 h dataset included 11 genes from the bHLH family, which was the top affected gene family (Supplementary Table S4), including the proneural transcription factor NEUROD1, which is critical for the differentiation of otic neurons as well as other bHLH factors associated with neurogenesis (*TCF3*, *TCF12*, *ID2*, *ID3*, *SIM1*, and *HES1*, *HES4*, *HES5.1*, *HES5.2*, *HES5.3*, *HEY1*). In contrast, the Notch LOF 24 h dataset contained genes associated with general cell biological or metabolic processes (Supplementary Table S5). The Toppgene pathway enrichment analysis for Notch LOF 6 h dataset revealed several matches for Notch signalling among the dysregulated pathways across all cross-referenced databases (Supplementary Table S4): Biocyc, KEGG, Reactome, the Pathway Interaction Database, Gene Set Enrichment Analysis (GSEA), Panther Classification System, The Rat Genome Database (RGD). On the other hand, Notch LOF 24 h dataset had only two matches for Notch signalling among significantly dysregulated pathways in Reactome and Panther databases (Supplementary Table S5). The marked differences in the 6 and 24 h transcriptional responses suggest that Notch inhibition triggers a rapid commitment of neurosensory progenitors to a neurogenic fate; however, after 24 h, it is possible that the neurosensory progenitor pool is depleted, due to excess formation of otic neurons, and that Notch inhibition regulates another set of non-neurogenic genes in the remaining otic cells.

The analysis of the enriched transcription factors binding sites (TFBS) showed that only 8 out of the top 50 TFBS were present in both LOF datasets: LEF1 (CTTTGT, V$LEF1 Q2), FOXO4 (TTGTTT, V$FOXO4 01), E12 (CAGGTG, V$E12 Q6), NFAT (TGGAAA, V$NFAT Q4), MAZ (GGGAGGRR, V$MAZ Q6), KDM7A Target Genes, and two unknown transcription factors with AACTTT and CTGCAGY binding motifs (Supplementary Tables S4, S5). Interestingly, the motif V$LEF1 Q2 was one of the most significantly enriched TFBS site in both datasets (*p* = 4.45e-15 in LOF 6 h; *p* = 4.73e-07 in LOF 24 h), with 51 genes from Notch LOF 6 h (Supplementary Figure S1; Supplementary Table S4) and 34 genes in Notch LOF 24 h (Supplementary Table S5). LEF1 is a member of the TCF/LEF transcription factor family and an essential component of the Wnt signalling cascade. Although these analyses were conducted using the human orthologues of the chicken genes, they suggest that some of the early and late transcriptional targets of Notch activity may be co-regulated by the Wnt pathway.

### 2.3 Inhibition of Wnt signalling regulates the expression of genes associated with neurogenesis, dorso-ventral specification and epithelio-mesenchymal differentiation

**FIGURE 4:**
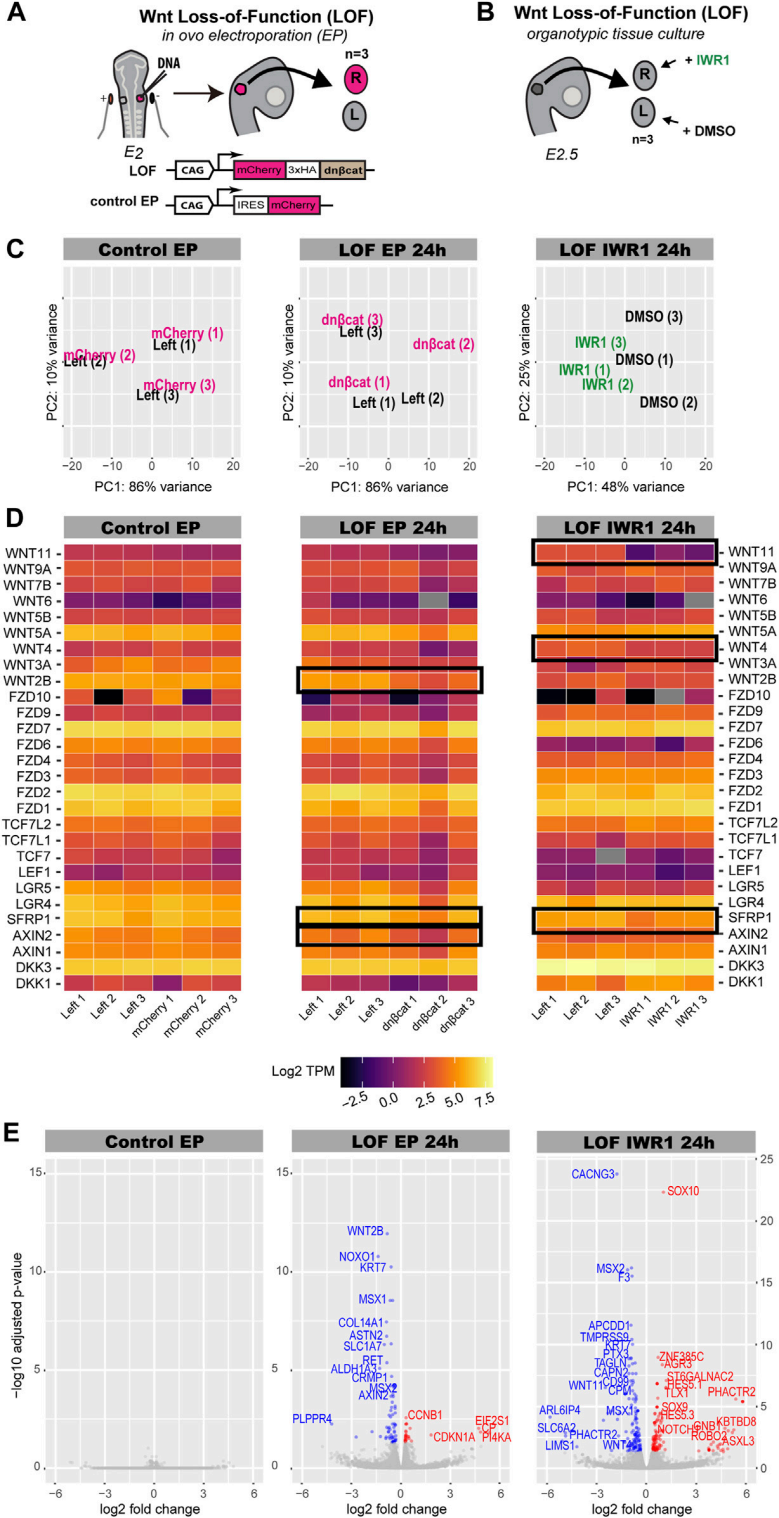
Characterisation of Wnt dataset. **(A,B)** Schematics of experimental design for Wnt inhibition by in ovo electroporation (Wnt LOF EP 24 h) and pharmacological treatment with IWR-1 (Wnt LOF IWR1 24 h). **(A)** Right otic cups of E2 chickens were electroporated with either a Tol2 construct overexpressing a dominant negative form of β-catenin (Wnt LOF EP 24 h) or a Tol2 control construct coding for mCherry (Control EP). Only three pairs of well transfected otocysts were taken for RNA-sequencing. **(B)** Three right otic cups were dissected from E2.5 chicken embryos and incubated in media containing IWR-1 for 24 h, while their left counterparts in media enriched with DMSO. **(C)** PCA plot showing distribution of treated otocysts vs. control samples in the Control EP, Wnt LOF EP 24 h and Wnt LOF IWR1 24 h groups. **(D)** Heatmap (log TPM) for Wnt signalling elements detected by bulk RNA-seq in chicken otocyst in all three datasets. High levels of gene expression are indicated by light yellow colour and low levels by dark purple, whereas grey marks genes without expression. Note that AXIN2 and WNT2B have decreased their expression in the ears transfected with dominant negative form of β-catenin compared to control left counterparts in the Wnt LOF EP 24 h group. **(E)** Volcano plots of Wnt LOF EP 24 h and Wnt LOF IWR1 24 h datasets with genes downregulated shown in blue and upregulated in red. In contrast, Control EP does not have significantly upregulated or downregulated genes. Of note, transcripts with particularly low q-value, CYP1A1 from Wnt LOF IWR1 24 h and LDHA and HTR1A from Wnt LOF EP 24 h, were not included in the volcano plots.

Next, we investigated the potential transcriptional targets of Wnt signalling during prosensory specification using two different approaches to inhibit Wnt activity (Żak and Daudet, 2021). Firstly, we electroporated chicken otic cups with a truncated form of β-catenin lacking the N- and C-terminus responsible for transcriptional activity (DNBCAT), which acts like a dominant-negative protein and inhibits Wnt signalling (Wnt LOF EP 24 h) (Figure 4A) (Żak and Daudet, 2021). The control group was electroporated with a plasmid driving expression of a monomeric red fluorescent protein (mCherry, Control EP) (Figure 4A). At 24 h post-electroporation, we collected left (untransfected) and right (transfected) otocysts from 3 embryos for both conditions. Secondly, we used a pharmacological inhibition of Wnt activity using IWR-1 (Wnt LOF IWR1 24 h) (Figure 4B), a tankyrase inhibitor that stabilises AXIN2, a member of the β-catenin destruction complex (Chen et al., 2009). For three E2.5 embryos, each right otic cup was incubated for 24 h in medium containing 300 μM IWR-1, while its left counterpart was maintained in medium containing DMSO at a matching 0.6% concentration as a control.

The PCA plot for the control electroporation (Control EP) showed no separation between left (control) and right (transfected) otocysts of each embryo (Figure 4C). Similarly, there was no clear separation of the Wnt LOF EP 24 h otocysts according to condition, (Figure 4C), suggesting little impact on the overall gene expression profile of transfected otocysts. In contrast, the PCA plot for Wnt LOF IWR1 24 h dataset showed a good separation of IWR-1 treated samples from the control DMSO treated samples along the PC1 (48% variance) (Figure 4C).

To assess the potential changes in Wnt signalling in response to our treatments, we analysed the expression levels of specific Wnt components (receptor, ligands, effectors, and modulators). Heatmaps of the abundance of log TPM for selected transcripts were generated for control EP, Wnt LOF EP 24 h and Wnt LOF IWR1 24 h groups. Mapping to the reference chicken genome with Kallisto (using default settings) detected 27 genes associated with Wnt signalling (Figure 4D, raw and log TPM values in Supplementary Table S1). Among these were the Wnt signalling modulators *DKK1*, *DKK3*, *LGR4*, *LGR5*, *AXIN1*, *AXIN2*, *SFRP1* (Figure 4D). All the Wnt pathway transcriptional effectors (*LEF1*, *TCF7*, *TCF7L1* and *TCF7L2*) as well as several receptors from the Frizzled family (*FZD1*, *FZD2*, *FZD3*, *FZD4*, *FZD6*, *FZD7*, *FZD9*, and *FZD10*) and Wnt ligands (*WNT2B*, *WNT3A*, *WNT4*, *WNT5A*, *WNT5B*, *WNT6*, *WNT7B*, *WNT9A*, *WNT11*) (Figure 4D) were detected. Out of these genes, *SFRP1*, *WNT2B* and *AXIN2* from the Wnt LOF EP 24 h and *SFRP1*, *WNT4* and *WNT11* from Wnt LOF IWR1 24 h were significantly regulated (q-value < 0.05) (Figure 4D).

**FIGURE 5:**
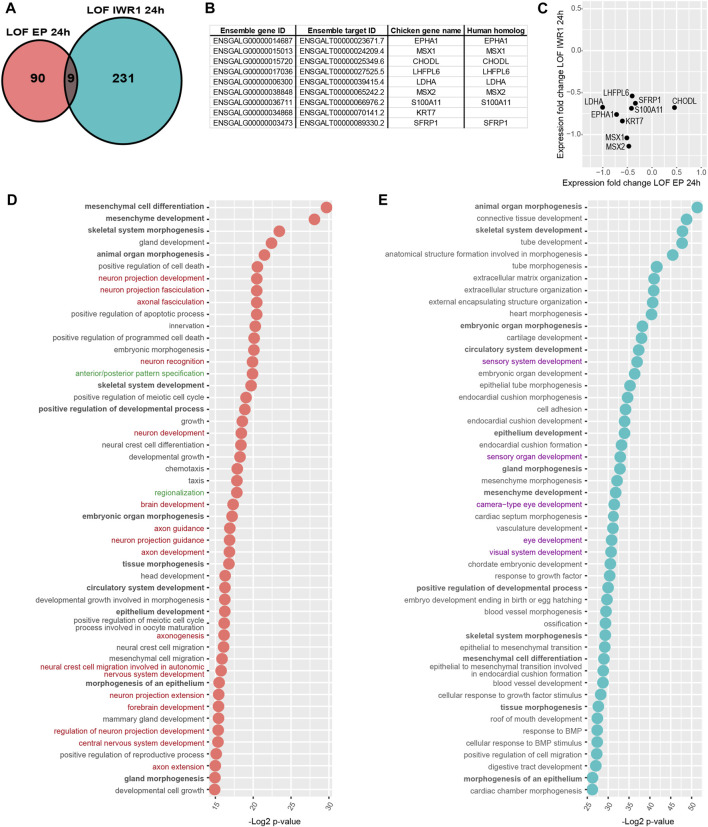
Comparison of Wnt LOF EP 24 h and Wnt LOF IWR1 24 h datasets. **(A)** Ven diagram showing that only 9 genes were common for the two datasets. **(B)** A table listing chicken Ensamble gene and target IDs for these 9 genes as well as their names and their human homolog names. **(C)** A plot showing the direction of changes in the expression of shared genes in the two Wnt datasets. **(D,E)** A comparison of top 50 GO biological function of Wnt LOF EP 24 h and Wnt LOF IWR1 24 h groups. The datasets share 12 biological functions (in bold), but only Wnt LOF EP 24 h has 16 functions associated with neurogenesis and neuron formation (in red) and 2 with regionalisation and body axis specification (in green). In contrast, Wnt LOF IWR1 24 h dataset **(E)** has genes implicated in 5 Go functions linked to eye and sensory organ development (in purple).

The control electroporation dataset did not have any significantly DE gene (q-value < 0.05, Figure 5E). In contrast, the Wnt LOF EP 24 h dataset contained 30 upregulated and 69 downregulated genes (Figure 4E), whilst the Wnt LOF IWR1 24 h dataset contained 109 upregulated and 132 downregulated genes (Figure 4E). Among the upregulated genes were the NOTCH1 receptor as well as proneural transcription factors, which are known direct target genes and canonical effectors of the Notch pathway.

However, the two Wnt LOF datasets shared only 9 candidate target genes (Figures 5A-C). Next, we used the ToppGene platform to compare the two Wnt datasets. Among top 50 significantly enriched biological functions, 12 were shared by Wnt LOF EP 24 h and Wnt LOF IWR1 24 h groups and were implicated with developmental processes and organ morphogenesis. Wnt LOF EP 24 h included 16 functions associated with neurogenesis and formation of neuronal tissue, and 2 functions related to regionalisation and body axis specification (Figure 5D; Supplementary Table S6). Additionally, among the top 200 significantly enriched GO functions were also 2 related to the regulation of the Wnt signalling pathway involved in dorsal/ventral axis specification. These bioinformatics results are in line with our recent findings showing that Wnt signalling regulates neurosensory specification along the dorso-ventral axis of the inner ear (Żak and Daudet, 2021). In contrast, the top 50 significantly enriched biological functions for Wnt LOF IWR1 24 h group were primarily associated to embryonic development, organ morphogenesis, and extracellular matrix organization (Figure 5E; Supplementary Table S7). None of the top 50 biological function terms was specifically associated with neurogenesis, but 5 of them were associated with the development of eye and sensory organs. It is worth noting that Wnt signalling was not among the significantly enriched gene families or signalling pathways in Wnt LOF EP 24 h (Supplementary Table S6) or Wnt LOF IWR1 24 h datasets (Supplementary Table S7). Nevertheless, both datasets contained an over-representation of genes with TFBS for LEF1 (CTTTGT, V$LEF1 Q2 and CTTTGA V$Lef1 Q2), which could represent direct targets of Wnt signalling. Among the other over-represented TFBS present in both datasets as well as the Notch LOF datasets were, FOXO4 (TTGTTT, V$FOXO4 01), E12 (CAGGTG, V$E12 Q6), MAZ (GGGAGGRR, V$MAZ Q6), and unknown transcription factor with AACTTT binding motif (Supplementary Tables S6, S7). The two Wnt datasets also shared 3 more TFBS: AP4 (CAGCTG V$AP4 Q5), MAX (V$MAX 01), and USF (V$USF 02).

### 2.4 Testing the function of new candidate regulators of prosensory specification

Our analyses identified a large number of genes associated with “neurogenesis” processes that could potentially be regulated by Notch and/or Wnt activities during prosensory specification. We decided to further test the function of some of these genes by overexpression in the chicken otocyst. We selected several transcription factors, which are prime candidate regulators of cell specification and differentiation events: NEUROD1, MEIS1A, MEIS2A, EGR1, LHX1, LHX5, DLX5, TCF3, MSX1, MSX2, PROX1, BAMBI, SOX9, SOX10, TBX2, and TFAP2C (Table 1). Additionally, we tested the effects of overexpressing the Wnt ligands WNT4, WNT5A, WNT11, and two critical negative regulators of Wnt signalling, AXIN2 and APC (Table 1).

**TABLE 1:** A list of constructs used in the study.

Gene name	Name of construct	Backbone	Insert	Origin/References
TBX2	pTbx2	p3xFLAG-CMV	Full length mouse Tbx2	Colin Goding Prince et al. (2004)
MSX1	pMsx1	pCMV-2b	Full length mouse Msx1	Rena D'Souza Ogawa et al. (2006)
MSX2	pMsx2	pEGFP-C1	Full length human Msx2	Luc Willems Twizere et al. (2005)
SOX9	pSox9	pcDNA3.1	Full length mouse Sox9	Peter Koopman Schepers et al. (2003)
LHX5	pLhx5	pCMV-Tag2A	Full length mouse Lhx5	Kin Ming Kwan Lui et al. (2017)
MEIS1A	pMeis1a	MSCV-IRES-YFP	Full length mouse Meis1a	Thomas Oellerich Mohr et al. (2017)
NEUROD1	pNeuroD1	pCAG	Full length mouse NeuroD1	Mark Emerson Patoori et al. (2020)
PROX1	pProx1	pEGFP-C1	Full length human Prox1	Panos Politis Kaltezioti et al. (2010)
EGR1	pEgr1	pcDNA3.1	Full length mouse Egr1	Addgene (ID#11729) Eileen Adamson Yu et al. (2004)
WNT11	pWnt11	pcDNA3.2	Full length human Wnt11	Addgene (ID#35922) Marian Waterman Najdi et al. (2012)
WNT4	pWnt4	RACS	Full length mouse Wnt4	Addgene (ID#13937) Cliff Tabin Hartmann and Tabin (2000)
TCF3	pTcf3	pcDNA3.1	Full length mouse Tcf3	Sergei Y. Sokol Hikasa et al. (2010)
Control	T2-mEGFP	Tol2	Membrane-localized EGFP	Nicolas Daudet Żak and Daudet (2021)
AXIN2	pAxin2	pCS2+	Full length mouse Axin2	Addgene (ID# 21279) Frank Costantini Jho et al. (2002)
LHX1	pLhx1	pcDNA3.1	Full length mouse Lhx1	Satchidananda Panda Hatori et al. (2014)
BAMBI	pBambi	pCMV5	Full length human Bambi	Ye-Guang Chen Lin et al. (2008)
DLX5	pDlx5	pcDNA3.1	Full length mouse Dlx5	Hyun-Mo Ryoo Lee et al. (2003)
SOX10	pSox10	pcDNA3.1	Full length human Sox10	Veronique Lefebvre Haseeb and Lefebvre (2019)
WNT5A	pWnt5a	pcDNA3.2	Full length human Wnt5a	Addgene (ID# 35930) Marian Waterman Najdi et al. (2012)
APC	pApc	pCMV-Neo-Bam	Full length human Apc	Addgene (ID# 16507) Bert Vogelstein Morin et al. (1997)
DNBCAT	DNBcat	Tol2	Membrane-localized Cherry; 2A self-cleaving peptide; triple HA-tagged truncated form of *Xenopus* β-catenin	Magdalena Żak Żak and Daudet, (2021)
NICD1	pNICD1-mRFP1	pCAG	HA-tagged chicken Notch1 intracellular domain; IRES; mRFP1	Nicolas Daudet Chrysostomou et al. (2012)
Cherry	T2-mCherry	Tol2	IRES: Membrane-localized Cherry	Magdalena Żak Żak and Daudet (2021)
mRFP1	mRFP1	pCAG	IRES; mRFP1	This study

**FIGURE 6:**
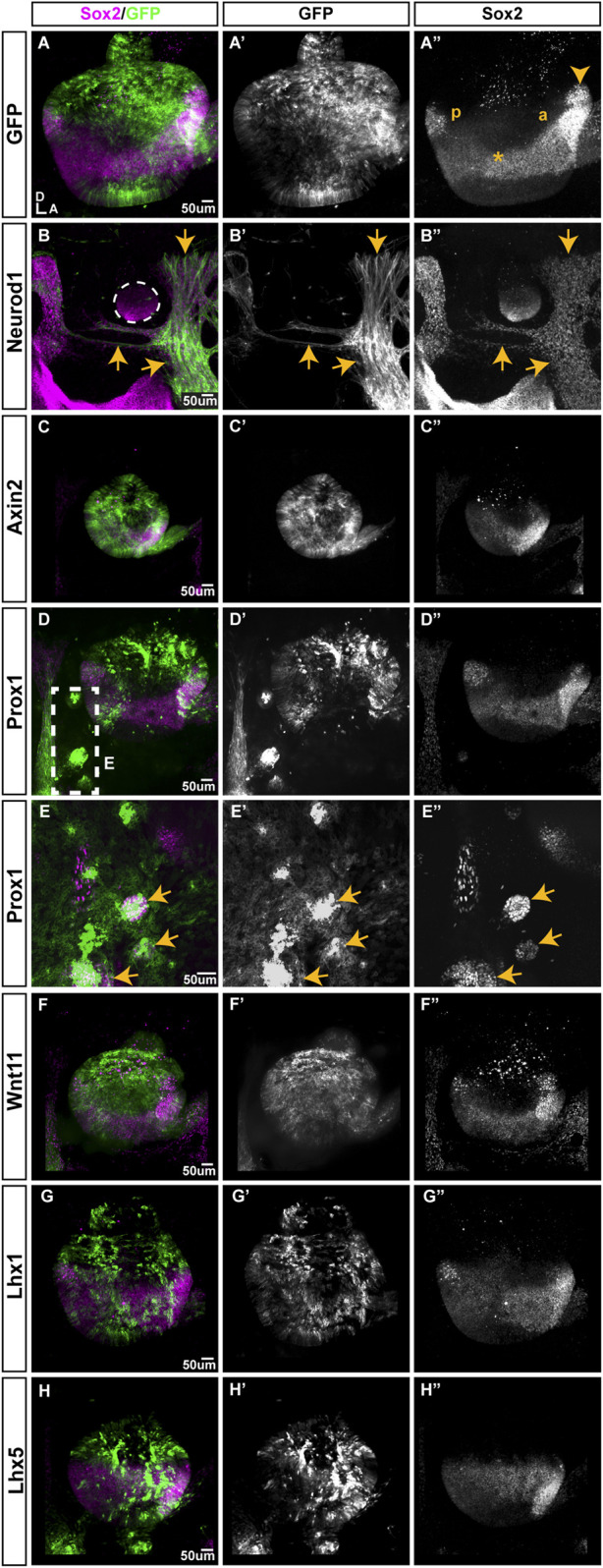
The effects of overexpression of selected genes on prosensory formation in the developing chicken inner ear. All samples were transfected at E2.5, collected 2 days post-EP and immunostained for SOX2. **(A)** Whole-mount view of an otocyst transfected with the T2-mEGFP plasmid (control); the anterior (a) and posterior (p) domains are visible, with a weaker SOX2-expressing domain extending between them (*). The prospective anterior crista has begun its segregation (arrowhead). B-I” Samples co-transfected with T2-mEGFP and either NEUROD1, AXIN2, PROX1, WNT11, LHX1, or LHX5. **(B)**. The NEUROD1-transfected cells (arrows) are GFP-positive, have neuronal-like processes and are intermingled with SOX2 expressing-cells around a tiny otocyst (outlined). Note the absence of GFP expression within the otocyst itself. **(C)**. AXIN2 transfected otocysts are very small; GFP-positive cells are located within both SOX2-expressing and non-expressing domains and include some delaminated otic neurons (arrows). **(D,E)**. PROX1-overexpression does not affect otocyst morphology, but some GFP-positive cells form SOX2-expressing clusters outside of the otocyst (box viewed at a higher magnification in E, arrows indicate ectopic SOX2-expressing patches). **(F–H)**. Otocysts transfected with WNT11, LHX1, or LHX5 have a relatively normal appearance and SOX2 expression pattern.

To mark transfected regions, plasmids expressing our genes of interest were co-electroporated with a plasmid encoding GFP. Transfected ears were collected 2 days post-EP and stained for the otic prosensory domain marker SOX2. We looked for ectopic or loss of SOX2 staining within the prosensory domains and abnormalities in the formation of otic neurons. Control experiments were performed with a GFP expression plasmid. Control ears (*n* = 4/4) developed normally forming posterior and anterior prosensory domains (occasionally including a well-segregated anterior crista) with elevated SOX2 expression and a sensory-competent domain with lower SOX2 expression stretching between them (Figures 6A–A”). Otocysts transfected with NEUROD1 (*n* = 4/4) (Figures 6B–B”) were very small in size, devoid of GFP expression, but surrounded by a large number of GFP-positive neuronal processes (Figures 6B–B”). Inside these small otocysts remained a population of SOX2-positive cells that did not form distinct prosensory domains. After overexpression of AXIN2 (Figures 6C–C”), WNT11 (Figures 6F–F”), LHX1 (Figures 6G–G”), LHX5 (Figures 6H–H”), the otocysts appeared to be smaller compared to the control ears and their prosensory domains seemed to be underdeveloped (*n* = 4/4), but otic neurons formed normally. Otocysts transfected with PROX1 had a normal appearance with posterior and anterior prosensory domains and otic neurons (*n* = 4/4) (Figures 6D–D”). However, the overexpression of PROX1 in the tissue surrounding the otocyst triggered formation of ectopic SOX2 domains (*n* = 2/4) (Figures 6E–E”). Overexpression of APC, WNT4, WNT5A, SOX9, SOX10, TBX2, DLX5, MSX1, MSX2, BAMBI, TCF3, MEIS1A, MEIS2A, and EGR1 (Supplementary Figure S2) did not trigger any changes in the formation of the otocyst and SOX2-expressing domains.

## 3 Discussion

In this study we aimed to find new regulators of neurosensory and prosensory specification in the embryonic chicken otocyst. Using pharmacological treatment *in vitro* and genetic manipulation *in ovo*, we modulated the Notch and Wnt signalling pathways and analysed the resulting changes in gene expression using RNA-Seq. The assessment of PCA results revealed an important variability within and between the datasets. In the Notch GOF and Wnt LOF EP datasets, individual samples did not cluster very well according to the treatment conditions and a relatively small number of differentially expressed genes were identified. This suggests that *in ovo* electroporation of the NICD1 or DNBCAT constructs induced relatively modest changes in gene expression in comparison to the other sources of inter-sample variability, in particular the inherent genetic differences between individual chicken embryos, which are not inbred animals. Further sources of variability could include differences in the developmental stages and the extent of electroporation, despite a careful selection of stage-matched embryos with well-transfected ears.

In comparison, the PCA of the Notch LOF 6 and 24 h datasets, relying on *in vitro* administration of GSI, showed a better clustering of individual samples according to treatment. Furthermore, we detected strong changes in the expression of known targets of Notch signalling from HES/HEY family in the Notch LOF 6 h and LOF 24 h datasets, confirming an efficient inhibition of Notch signalling by GSI. The Wnt LOF datasets obtained from otocysts treated with IWR-1 *in vitro* also showed a better clustering of the samples according to treatment and a much greater number of differentially expressed genes compared to DNBCAT-electroporated samples.

In conclusion, these results showed that *in vitro* pharmacological treatments induce stronger and more reproducible transcriptional responses than *in ovo* electroporation of chicken otocysts. This is most likely a direct consequence of the mosaicism and variability of plasmid transfection by electroporation, which is a clear limitation for whole-otocyst RNA-Seq analyses, even in seemingly well-transfected samples. Future experiments relying on *in ovo* transfection of plasmid DNA should therefore include an additional fluorescence-activated cell sorting step or be combined to single-cell RNA-seq analyses to overcome the mosaicism of transfection. Finally, none of the treatments detected significant changes in the expression levels of JAG1 or SOX2, two prosensory factors which have been shown in previous studies to be regulated by Notch and Wnt activities. Whilst this may point at some form of post-transcriptional regulation of these factors, it is also possible that the bulk RNA-Seq approach is fairly limited in terms of sensitivity and a greater number of samples might have been needed to increase the statistical power of our analyses.

### 3.1 Blocking Notch signalling induces a rapid dysregulation of genes associated with neurosensory differentiation

The comparison of two durations of GSI treatment, Notch LOF 6 h and LOF 24 h, revealed striking changes in the set of differentially expressed genes, which were associated with distinct classes of biological processes. In fact, only 17 genes were shared between the 6 and 24 h LOF datasets; among these were 4 Notch effectors of the HES family (*HES 5.1*, *HES 5.2*, *HES5.3*, *HES4*), whose downregulation confirmed a persistent blockade of Notch activity by GSI. In accordance with the role of Notch lateral inhibition in preventing excessive neuroblast formation in the otocyst (Haddon et al., 1998; Abello et al., 2007; Daudet et al., 2007), the loss of Notch activity in the LOF 6 h dataset affected the expression of several genes implicated in otic neurogenesis. Among these were the genes encoding NEUROD1, a proneural bHLH transcription factor essential for audiovestibular neuronal differentiation (Liu et al., 2000; Filova et al., 2022) and the Notch ligand DLL1, mediating lateral inhibition in otic neuroblasts (Adam et al., 1998; Daudet et al., 2007; Daudet and Żak, 2020). As expected from a failure of lateral inhibition, the expression of both *NEUROD1* and *DLL1* was upregulated in the 6 h LOF dataset. Other dysregulated genes associated to the differentiation of otic neurons and sensory organs included *PROX1* (upregulated) (Stone et al., 2003; Bermingham-McDonogh et al., 2006; Fritzsch et al., 2010; Nishimura et al., 2017), *FGF10* and *FGF20* (downregulated) (Pauley et al., 2003; Hayashi et al., 2008; Munnamalai et al., 2012; Urness et al., 2015), *FGF3* (upregulated) (Zelarayan et al., 2007; Olaya-Sanchez et al., 2017), *BMP4* (downregulated) (Wu and Oh, 1996; Morsli et al., 1998; Chang et al., 2008). The LIM-homeodomain transcription factor LMX1B, which plays a critical role in the segregation of sensory organs (Nichols et al., 2008; Koo et al., 2009), was upregulated in the Notch LOF 6 h dataset, which is consistent with previous studies showing that Notch activity represses *LMX1B* expression in the chicken otocyst. Finally, there was an overrepresentation of dysregulated genes with E-box motifs (CAGGTG; Supplementary Table S4), which are binding sites for the ubiquitous E12/E47 bHLH proteins associated to the tissue-specific proneural bHLH transcription factors (Jennings et al., 1999; de Martin et al., 2021). Altogether, these results show that the blockade of Notch activity induces a rapid transcriptional response associated to neurosensory differentiation.

In contrast, the genes affected by the prolonged loss of Notch activity in the LOF 24 h dataset were primarily associated with general cellular biology processes such as protein translation and trafficking as well as various catabolic processes. Furthermore, the proneural gene *NEUROD1* was no longer differentially expressed in the LOF 24 h dataset and E-box motifs were not over-represented among the DE genes. The differences with the shorter treatment could result from the more advanced developmental stage of the otocyst as well as the fact that at 24 h post-blockade, most neurosensory precursors committed to a neuronal fate have presumably delaminated from the otocyst. This could explain the reduction in expression levels of neurogenesis-associated genes within the otic samples processed for RNA-Seq.

### 3.2 Inhibition of Wnt signalling by IWR1 or DNBCAT produces different transcriptional responses

Our recent work showed that the Wnt signalling pathway forms an activity gradient along the dorso-ventral axis of the otocyst to control sensory organ formation and neurogenesis in a dose-dependent manner (Żak and Daudet, 2021). To identify targets of Wnt signalling in this context we used two distinct approaches to block Wnt activity in the otocyst. Pharmacological treatment with IWR-1 increases the intracellular levels of AXIN2 protein, thereby promoting formation of a destruction complex and degradation of beta-catenin (Chen et al., 2009), whereas overexpression of truncated form of beta-catenin lacking transcriptional activity and acting as a dominant-negative form prevents endogenous beta-catenin from activating its transcriptional targets (Żak and Daudet, 2021). We have shown that both approaches can reduce the activity of a fluorescent Wnt reporter (comprising 5 TCF/LEF binding sites) in the chicken inner ear (Żak and Daudet, 2021).

The Wnt signalling pathway does not have a set of context-independent core target genes and the ear-specific Wnt target genes are still unclear but, both Wnt LOF datasets contained an over-representation of DE genes with TFBS for LEF1, suggesting some alteration of Wnt activity. Among the 9 shared genes between the Wnt LOF IWR1 24 h and Wnt LOF EP 24 datasets were *MSX1* and *MSX2*, which are expressed in the vestibular system and endolymphatic duct (Wu and Oh, 1996; Pujades et al., 2006) where high levels of Wnt activity are present. Interestingly, Wnt signalling positively regulates expression of *MSX1* and *MSX2* genes in other systems and both were downregulated in our LOF datasets (Willert et al., 2002; Szemes et al., 2020). Two other candidate targets of Wnt signalling are SFRP1 and AXIN2, which are negative regulators of Wnt activity (Finch et al., 1997; Behrens et al., 1998; Seidensticker and Behrens, 2000). AXIN2 is positively regulated by Wnt signalling in other tissues (Jho et al., 2002) and was significantly downregulated in the Wnt LOF EP24 h dataset. SFRP1, a soluble inhibitor of Wnt signalling that binds to Wnt ligands or Frizzled receptors in the extracellular compartment (Lin et al., 1997; Bafico et al., 1999), was downregulated in both LOF datasets and its paralogue SFRP2 is a known Wnt target gene (Lescher et al., 1998) in other tissues. These results suggests that both IWR-1 and DNBCAT overexpression affect Wnt signalling and that *MSX1*, *MSX2*, *SFRP1*, and *AXIN2* may be Wnt targets in the inner ear.

Similarly to our Notch datasets, we identified more DE genes through pharmacological treatment than after overexpression of DNBCAT. The biological functions in both Wnt datasets were associated with morphogenesis, organ development and neurosensory tissue formation, but only 12 out of the top 50 functional annotations were common. This relatively low level of overlap may be due to the two very different approaches (*in ovo* and *in vitro*) targeting different elements of the Wnt signalling cascade. From the bioinformatics analysis, the Wnt LOF EP 24 h dataset appeared better aligned with our current understanding of the role of Wnt signalling during early inner ear development. Indeed, it highlighted several biological functions related to neurogenesis as well as antero-posterior (A-P) and dorso-ventral (D-V) axis specification. This is in line with previous studies demonstrating the roles of Wnt signalling in establishing the D-V patterning of the otocyst and its neurosensory territories (Stevens et al., 2003; Riccomagno et al., 2005; Noda et al., 2012; Rakowiecki and Epstein, 2013; Żak and Daudet, 2021). Our data suggest a number of Wnt signalling components could contribute to the establishment of the D-V gradient of Wnt activity. Wnt antagonists such as SFRP1, which is expressed in the ventral region of the inner ear (Sienknecht and Fekete, 2008; Sienknecht and Fekete, 2009), and AXIN2 could be part of a negative feedback loop progressively reducing the levels of Wnt activity along the D-V axis. Furthermore, three Wnt ligands (WNT2B, WNT4 and WNT11) were downregulated in at least one of the LOF conditions, suggesting some form of positive feedback dependent on Wnt activity could regulate their expression. The Wnt pathway is known to form such feedback mechanisms in other systems through Wnt-mediated expression of ligands and inhibitors (Lustig et al., 2002; Stuckemann et al., 2017) and it will be important to further investigate their contribution to inner ear patterning.

### 3.3 The endogenous program of neurosensory specification is robust

With the aim to identify new genes regulating neurosensory specification, we overexpressed in the chicken otocyst 21 of the genes identified in our Wnt and Notch datasets encoding either transcription factors, which are prime candidate regulators of cell differentiation, or components of the Wnt pathway. Disappointingly, only two transcription factors, NEUROD1 and PROX1, produced a noticeable phenotype 48 h after *in ovo* EP. NEUROD1-overexpressing cells formed neuron-like cells which delaminated from and surrounded the otocyst, confirming its importance for neuronal differentiation (Liu et al., 2000; Kim et al., 2001). PROX1-overexpressing cells formed clusters with ectopic SOX2 expression, but only outside of the otocyst. The significance of this result is unclear, although previous studies have shown that PROX1 overexpression can induce the formation of neurons in the CNS (Karalay et al., 2011; Iwano et al., 2012; Kaltezioti et al., 2021). AXIN2 overexpression drastically reduced the size of the otocyst, possibly reflecting an effect on cell proliferation or survival, but did not affect the spatial pattern of SOX2 expression. None of the other Wnt components induced a phenotype. These results suggest that these factors do not regulate SOX2 expression, or may do so in cooperation with other signals. They also indicate that the endogenous program of neurosensory differentiation is remarkably robust and only a small number of key transcriptional factors, such as proneural genes, SOX2 and LMX1A (Neves et al., 2011; Mann et al., 2017), might be able to override the signals driving sensory versus non-sensory cell differentiation.

## 4 Conclusion

Our analyses of the transcriptional responses to the modulation of Wnt and Notch activities have provided new insights into the complex nature of the effectors and targets of these pathways in the early developing inner ear. They have also uncovered a list of genes that might be co-regulated by these two pathways. Our functional screening suggest that the manipulation of a single transcription factors is not as efficient to influence otic neurosensory cell differentiation as the genetic or pharmacological modulation of either the Wnt or the Notch pathway (Daudet and Lewis, 2005; Riccomagno et al., 2005; Daudet et al., 2007; Rakowiecki and Epstein, 2013; Daudet and Żak, 2020; Żak and Daudet, 2021). This could be particularly relevant to the design of strategies to generate inner ear sensory cells through genetic reprogramming, in particular from embryonic stem cells of for hearing loss therapies. Finally, one alternative approach to either type of manipulation might be to target some of the immediate effectors of both pathways. Not much is known about the expression and function of individual LEF/TCF factors in the developing inner ear and our analyses suggest that Lef1 is of particular interest, given the abundance of Notch target genes with LEF1 TFBS in their promoter regions. Further studies are needed to clarify its role during the formation of the Wnt activity gradient and prosensory specification in the otocyst.

## 5 Materials and methods

### 5.1 Animals

Fertilised White Leghorn chicken (*Gallus gallus*) eggs were obtained from Henry Stewart UK and incubated at 37.8°C for the designated times. Embryonic stages refer to embryonic days (E), with E1 corresponding to 24 h of incubation or to Hamburger and Hamilton stages (Hamburger and Hamilton, 1951). All procedures were approved by University College London local Ethics Committee and by the UK Home Office.

### 5.2 *In ovo* electroporation

Electroporation of the otic placode/cup of E2 chick embryos (stage HH 10–14) was performed using a BTX ECM 830 Electro Square Porator as previously described (Freeman et al., 2012). The total concentration of plasmid DNA ranged for each set of experiments between 0.5 and 1 μg/μl. Unless otherwise specified, for the gain of function experiments a minimum number of 4 well transfected samples were examined for each experimental condition (Figure 1A). For the bulk RNA-seq analysis, embryos were collected and examined with a fluorescent dissecting stereomicroscope. Those with the most intense and widespread fluorescence signal within the otocyst (Figure 1A) were selected for analysis. Both left (non-transfected) and right (transfected) otocysts were dissected from each embryo in ice-cold L-15 medium (Leibovitz), cleaned from surrounding mesenchyme and immediately processed for total RNA isolation. The expression of mesenchymal markers was assessed for all datasets and included in Supplementary Table S1.

### 5.3 Plasmids

The plasmids used in this study and their origin are described in the Table 1. New constructs were generated using the In-Fusion HD Cloning Kit (Takarabio).

### 5.4 Organotypic tissue culture

Both left and right ears were dissected from chicken embryos aged E2.5 in ice-cold L-15 medium, cleaned from surrounding mesenchyme and individually incubated as free-floating cultures. Left ears were incubated in media enriched with DMSO (control conditions) and right ears were treated with γ-secretase inhibitor LY411575 (10 µM, Notch LOF datasets) or IWR-1 (300 µM, Wnt LOF datasets) for 6 or 24 h.

### 5.5 RNA-sequencing and bioinformatics analysis

Total RNA was extracted from each individual otocysts using the RNAqueous™-Micro Total RNA Isolation Kit (Ambion) according to the manufacturers protocol. The quality of isolated RNA was tested using Agilent 2200 Tapestation and only samples with a value of RNA integrity number of at least 9 were used for library preparation by UCL Genomics. The SMART-Seq v4 Ultra Low Input RNA Kit (Clontech Laboratories, Inc.) was used to generate cDNA libraries using 10 cycles of PCR. cDNA was checked for integrity and quantity on the Agilent Bioanalyser using the High Sensitivity DNA kit, and 200 pg of cDNA was then converted to sequencing library using the Nextera XT DNA protocol (Illumina, San Diego, United States). Samples were sequenced 43 bp paired-end read and ∼16M reads per sample length on NextSeq 500 instrument (Illumina, San Diego, CA, United States). Run data were demultiplexed and converted into fastq files using Illumina’s bcl2fastq Conversion Software v2.19. Kallisto package (Bray et al., 2016) was used to map reads to a chicken reference genome (Gallus_gallus-6.0) and to quantify abundances of transcripts. Differentially expressed genes were identified using Sleuth package (Pimentel et al., 2017) by comparing treated samples with control ears from the same embryo. Genes with q-value (Wald test) lower than 0.05 were considered as significant. Functional annotations were downloaded from ENSEMBL using biomaRt and PCA plots were generated using DESeq2. The heatmaps were generated in R using logarithm (base 2) of TPM values. Signalling pathway enrichment, transcription factor binding site enrichment and Biological Function enrichment analysis were performed using Toppgene online tool with default settings and using the human orthologues of the chicken genes as input. Visualizations of gene networks were generated using Cytoscape. To distinguish between the expression of endogenous NOTCH1 and overexpressed NIDC1, we included in the reference genome two separate sequences for the chicken intracellular (NICD1) and extracellular domains of NOTCH1. Next, we compared the number of reads for intracellular (NICD1) and extracellular sequences of NOTCH1 gene in each sample and analysed changes in their expression levels between the treated and non-treated samples. The tpm results are included in Supplementary Table S1 tab “NICD1_EP_tpm” The original datasets are publicly available in GEO. For the Wnt LOF datasets please see https://www.ncbi.nlm.nih.gov/geo/query/acc.cgi?acc=GSE149310 and https://www.ncbi.nlm.nih.gov/geo/query/acc.cgi?&acc=GSE230083; for all Notch datasets https://www.ncbi.nlm.nih.gov/geo/query/acc.cgi?acc=GSE196999.

### 5.6 Immunohistochemistry

Chicken embryos (E3-E4) were collected, fixed for 1-h in 4% paraformaldehyde (PFA) in 0.1 M phosphate buffered saline (PBS), and processed for whole-mount immunostaining. The head was dissected along the midline, the hindbrain was removed, and the region surrounding the otocyst was only partially trimmed to facilitate orientation. Next, the tissue was permeabilized in PBS containing 0.3% Triton and 10% goat serum for 30 min at room temperature. Specimens were incubated with primary antibodies diluted in 0.1% Triton in PBS at 4°C overnight. On the next day, tissues were rinsed with PBS at room temperature and incubated with secondary antibodies diluted in 0.1% Triton and 10% goat serum at 4°C overnight. Afterward, tissues were again rinsed with PBS and mounted in Vectashield Antifade Mounting Medium (Vector laboratories). The following antibodies were used: mouse IgG1 monoclonal anti-Sox2 (BD Biosciences, San Jose, CA; 561469, 1:500). Secondary goat antibody conjugated to Alexa dye (1:1000) were obtained from Thermo Fischer Scientific (United Kingdom). Confocal stacks were acquired using a Zeiss LSM880 inverted confocal microscope, further processed with ImageJ and arranged in Adobe Illustrator.

## Data Availability

The datasets presented in this study can be found in online repositories. The names of the repository/repositories and accession number(s) can be found below: GEO, GSE149310 and GSE230083, GSE196999.

## Supplementary material

SUPPLEMENTARY FIGURE S1 Genes in Notch LOF 6 h and Notch LOF 24 h dataset identified as LEF1 targets genes. CDK6 and TCF12 (genes in black circle) are shared between the two datasets. The size of nodes represents q-value and the colour of node fill reflects the direction and intensity of changes in the expression.

SUPPLEMENTARY FIGURE S2 The overexpression of APC (A–A”), WNT4 (B-B”), WNT5A (C–C”), SOX9 (D–D”), SOX10 (E–E”), TBX2 (F–F”), DLX5 (G–G”), MSX1 (H–H”), MSX2 (I–I”), BAMBI (J–J”), TCF3 (K–K”), MEIS1A (L–L”), or MEIS2A (M–M”) does not affect the size of the inner ear or the SOX2 expression pattern.
