# Supplementary figures and images for: Regulation of otic neurosensory specification by Notch and Wnt signalling: insights from RNA-seq screenings in the embryonic chicken inner ear
Source: Front Cell Dev Biol. 2023 Oct 12;11:1245330. doi: 10.3389/fcell.2023.1245330 (PMC10600479; doi:10.3389/fcell.2023.1245330)

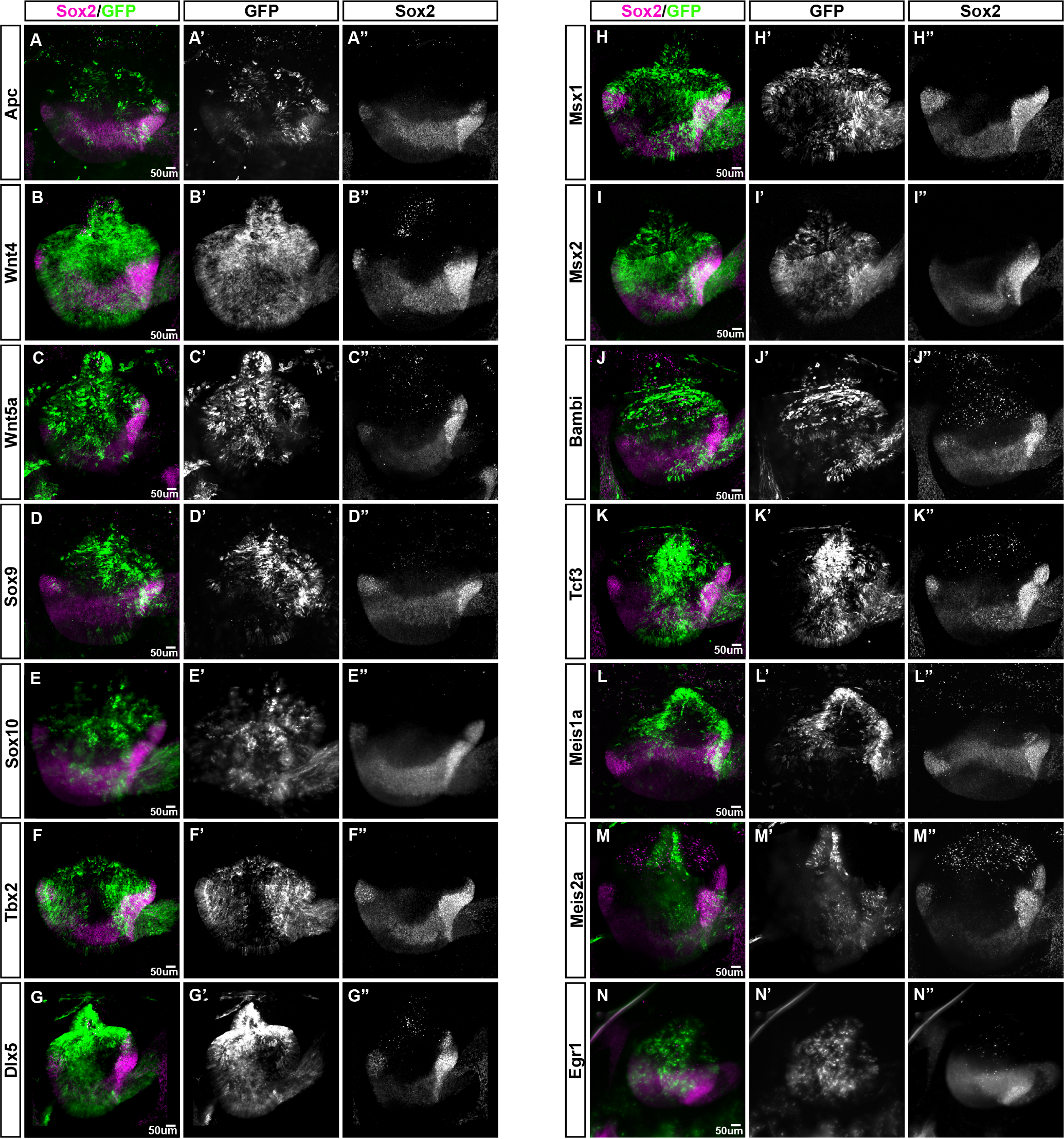

Supplement: Supplementary file 3 [file Presentation1.zip › Sup_Figure_2.tif]

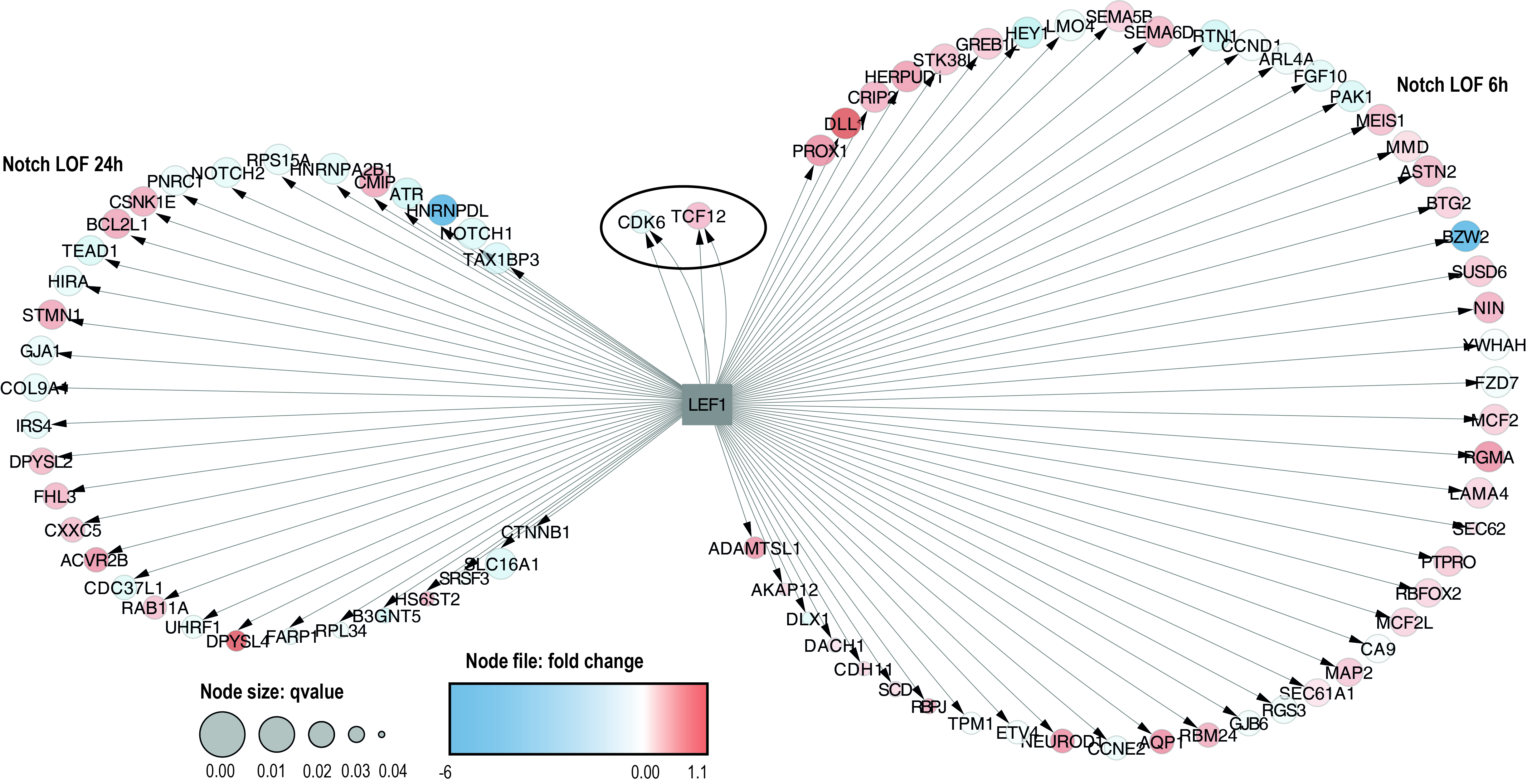

Supplement: Supplementary file 3 [file Presentation1.zip › Suppl Figure 1.tif]
